# Supplementary material for: Clinical Decision Support System to Detect the Occurrence of Ventilator-Associated Pneumonia in Pediatric Intensive Care
Source: Diagnostics (Basel). 2023 Sep 18;13(18):2983. doi: 10.3390/diagnostics13182983 (PMC10528404; doi:10.3390/diagnostics13182983)
Supplement: Supplementary file 1 [file diagnostics-13-02983-s001.zip › diagnostics-2601518-supplementary.pdf]

Data Cleaning

For ventilatory data such as inspired fraction of oxygen (FiO2), positive end-expiratory pressure (PEEP), mean airway pressure (MAwP), peak inspiratory pressure (PIP), *Freq.respi.total*), first zero values were considered as missing values. Then, the last available reported value was used to be replaced by the missing data for the upcoming event. In case of missing data at the beginning of the ICU stay, the first available data was used.

For any value missing in *Vol.C.r*, we set the corresponding *tidal volume* as missing as well. To handle the outliers, physiologically impossible values were replaced as follows. In case equal values for variables *PEEP* and *PIP* were observed, in *PIP* the value corresponding to the previous observation was used.

Segmenting variables in time blocks

At this stage, first time blocks of 6 hours were generated using the variable *time*.

For each patient total number of blocks of 6 hours was calculated. Then backward time blocks of 6 hours were created and added to the data set under the variable name *timeblocks*.

To better synchronize the data, avoid the impact of outliers and limit the missing data, for each continuous variable the median was calculated over each time block of 6 hours. For the categorical variable, subjective amount of respiratory tract secretion (Qt.Secretions), mode (the most frequently occurring category while ignoring the missing values) was selected.

Next step was to generate the data for the time blocks of 48 hours with the following variables: *Leucocytes, Neutrophiles, PCR,* inspired fraction of oxyge (FiO2), positive end-expiratory pressure (PEEP), mean airway pressure (MAwP), pulmonary dynamic compliance (compliance), minute ventilation, Saturation and inspired fraction of oxygen ratio (sf), Oxygenation and Saturation Index (osi), Qt.secretions . We decided to analyze observations over the time blocks of 48 hours to be as close as possible to the CDC criteria. For this step, we only considered the patients with at least 16 blocks of 6 hours of ICU stay. Since all infections happening during the first 48h of hospitalization cannot be considered as nosocomial infections, we removed observations for the first 48 hours for each patient. Moreover, patients with less than 4 days of invasive mechanical ventilation were removed because the clinical decision system was built to analyze time blocks of 48 hours.

For patients with VAP, we generated two separate sets of data. One corresponds to the observations from the last 48h of ICU stay when the VAP occurred, and another one for the time period before occurring VAP.

The procedure to generate the data set corresponding to the VAP events was as follows. We took the first and the last values in the last 48 hours of ICU stay, for the variables *Leuco- cytes, Neutrophiles, PCR,* and FiO2. For other variables, we considered the first and the last non-missing values, if there was any, in the last 48h time period. For each variable, we stored these values in different set of variables, one for the first value (Fvalue) and one for the last value (Lvalue). We also added another

set of variables (Delta) which compares Fvalue and Lvalue.

For the variables FiO2, PEEP and MAwP, we considered the actual differences (Delta=Lvalue-Fvalue). For *Leucocytes, Neutrophiles, PCR,* minute ventilation and osi, the relative changes (Delta=(Lvalue-Fvalue)/Fvalue), and for compliance and sf negative relative changes (Delta=(Fvalue-Lvalue)/Fvalue). To have finite values for Delta defined as relative changes, we replaced the zero value in Fvalue by the the non-zero Fvalue in the preceding time block. For Qt.secretions, we have 4 categories $\{0, 1, 2, 3\}$. Note that, in the absence of a reading, we considered that there was no secretion. So, the missing observations were replaced by "0". If Fvalue or Lvalue is 0 or 1, we set Fvalue and Lvalue to 1. Otherwise, we select the observed values Fvalue and Lvalue. The Delta, for Qt.secretions, would be the actual difference (Delta=Lvalue-Fvalue). We generated a separate data set to store the events for the time blocks of 48h before VAP occurs. We followed the same procedure in the previous step, except here we had Fvalue, Lvalue and Delta for each time block of 48h. These values were stored in backward time order in three separate columns. We also created a data set for the patients without VAP with the same method.

For further analysis, we considered two sets of variables. One containing the observations for Fvalue and another one corresponding to Delta for each variable.

Train-test split

To build a predictive model, we split the data in two sets. A train set to develop the model and a test set to validate the model.

In this step, we created two lists from the patients included in the three data sets generated in the section “Segmenting variables in time blocks”. One list for the patients with the VAP events (65 patients), and another list consist of patients in the data sets corresponding the time blocks without VAP (660 patients). Each of these lists were randomly split in train group and test group in a 70:30 ratio.

Using these two lists, we generated train and test data sets. Observations corresponding to the patients in the train group were stored in the train set, and observations for the patients in the test group were stored in the test set.

In both sets, the VAP event was assigned to the binary variable *VAP* (whether VAP occurred

or not; 1 or 0). The number of VAP events in train and test sets were 45 and 22, respectively. Note that, certain patients were intubated on multiple occasions. This leads to a greater total number of VAP events compared to the number of patients with the VAP.

Number of observations for the free of VAP events in the train and test sets were 1852 and 788, respectively. We recall that each line of observation in these data sets corresponds to the values in a time block of 48h.

Test and train sets consist of the patients' ID, observations for the variables *VAP*, as well as Fvalue and Delta for the variables FiO2, PEEP, MAwP, minute ventilation, sf, osi, compliance, and Qt.secretions.

Imputation

First, we identified the variables with missing values in both train and test sets.

We observed 2 missing values for Saturation and inspired fraction of oxygen ratio (sf) and Oxygenation and Saturation Index (osi) in the test set. For variables pulmonary compliance (compliance) and minute ventilation, the number of missing values in train and test sets were 935 and 439, respectively.

Missing Value imputation in train set was done by 'randomForest' (v4.6-14) with the function 'rfImpute'. The imputed values are the weighted average of the non-missing observations, where the weights are the proximities from randomForest. The missing values in each variable of the test set were replaced by the mean of imputed values for the same variable with missing values in the train set. In the case where variables were complete in train and not in

the test set, the missing values in the test set were replaced by the mean of the values in the train set. This process can be applied prospectively to new data.

Predictive models

The first implemented algorithm was Random Forest by 'randomForestSRC' (V2.9.3), with the function 'rfsrc'. The performance of this model is measured using the error rate. The number of tress (*ntree*) was set to the default number of 1000. In the outcome, the number of variables randomly sampled as candidates for splitting a node (*mtry*) was 4. To return the variable importance (VIMP) information, we set *importance="TRUE"* in the function.

Our data set had a structure of an imbalanced data where the proportion of the majority class (free of VAP) to the minority class (VAP) is much larger than one (the imbalanced ratio was about 38). Given this situation, we then fit a classifier with 'imbalanced' function using the balanced random forest method (BRF). BRF method under-samples the majority class (the class with the greater frequency) so that its cardinality matches that of the minority class. The performance of 'imbalanced.rfsrc' is measured using the Geometric Mean. The number of trees was set to 500.

We also used K-fold cross validation technique to get more information about our algorithm performance. The K-fold cross-validation splits the data into K equally sized parts (fold), and iteratively trains the model on K-1 folds and test it on the holdout Kth fold. We implemented 5- fold cross validation by caret package and using "train" function. The function "traincontrol" was used to specify the type of resampling (method="cv"). We fit both Stepwise Regression ("glmStepAIC") and Random Forest ("rf") models. For these models, accuracy was used to select the optimal model using the largest value. The final value for the number of trees was 500 and *mtry* was 2.

Finally, we applied Elastic net (EN) and weighted Elastic net (WEN) regularizations by caret package using 5-fold cross-validation. In the *trainControl* function, we used the method *"repeatedcv"* with a *"random"* search. In the train function the method *"glmnet"* was used and the tuning grid was set to 25 to fit the elastic net. In the weighted Elastic Net, we assigned weights 38 (the imbalanced

ratio) for VAP events and 1 for free of VAP events. AUC was used to select the optimal model using the largest value. The final values of the penalty coefficients used for the model were derived.

**Method**	**Function/method**	**Measure of Performance**	**Hyper-Parameter**
**RandomForest**	rfsrc	Error rate (overall error rate2.37%)	ntree=1000, mtry=4
**Imbalanced RF**	imbalanced(brf)	G-mean=0.74	ntree=500, mtry=4
**Stepwise.Reg. (5-fold CV)**	glmStepAIC	Accuracy=0.97	NA
**RandomForest (5-fold CV)**	rf	Accuracy= 0.98	ntree=500, mtry=2
**ElasticNet.Reg (5-fold CV)**	glmnet	AUC=0.83	α=0.019;λ=0.042
**Weighted ElasticNet (5-fold CV)**	glmnet	AUC= 0.83	α=0.58;λ=0.034

Per patient validation

In this step, we evaluated the final model on its capacity to correctly assess the infection status of patients over time.

Two approaches were applied: first, we looked at the accuracy of predictions by stratifying patients into subgroups. Then, we evaluated performance of the model over time.

The procedure is as follows.

Prediction results from the model

From the final model, chosen based on its performance, we generated a data set consist of 5 variables; the predicted classes (Pred), the predicted classes using the thresholds correspond to the levels of sensitivity 80% (Pred.th1), and 85% (Pred.th2), and variables *VAP* and *ID*.

Subgroup stratifications

Patients in the test group were divided into two subgroups. A group of patients for whom we had at most 3 time blocks of observations (G1), another group of patients with at least 4 time blocks of observations (G2). This classification was done separately for patients with VAP and without VAP. The number of patients with VAP for subgroups G1 and G2 are 13 and 9, respectively. For patients without VAP, we have 186 and 40.

Subgroups' validation based on the final model

The number of patients for whom we obtained accurate predictions (i.e. predicted class=observed VAP status) in class predictions Pred, Pred.th1 and Pred.th2 are 185, 165 and 157. The number of patients with inaccurate predictions (i.e. predicted class ≠ observed VAP status) over time, in each class predictions, are 7, 13 and 17, respectively.

We identified the patients for whom the predictions contained at least one error (i.e. there exists an observation where predicted class ≠ observed VAP status) for each subgroup G1 and G2. The global error rates were calculated for each subgroup and presented in supplemental table 1 (error prediction).

Model performance over time

In this step, we generated a data set that compares the observed value of VAP with its predicted value for all patients and in all time blocks, starting from the first time-block and progressively accounting for subsequent blocks. If predicted value of VAP status corresponds to the observed value over the time-period considered, we set the validation value as 1, otherwise as 0.

From this data set we computed false positive rates (FPR) and true positive rates (TPR) over time. We define

- FPR= number of patients with VAP status 0 and validation value 0 / number of patients with VAP status 0
- TPR= number of patients with VAP status 1 and validation value 1 / number of patients with VAP status 1
